# Individual and community-level determinants and spatial distribution of prenatal HIV test uptake in Ethiopia: Spatial and multilevel analysis

**DOI:** 10.3389/fpubh.2023.962539

**Published:** 2023-02-21

**Authors:** Nuhamin Tesfa Tsega, Daniel Gashaneh Belay, Melaku Hunie Asratie, Moges Gashaw, Mastewal Endalew, Fantu Mamo Aragaw

**Affiliations:** ^1^Department of Women's and Family Health, School of Midwifery, College of Medicine and Health Sciences, University of Gondar, Gondar, Ethiopia; ^2^Department of Human Anatomy, College of Medicine and Health Sciences, University of Gondar, Gondar, Ethiopia; ^3^Department of Epidemiology and Biostatistics, College of Medicine and Health Sciences, Institute of Public Health, University of Gondar, Gondar, Ethiopia; ^4^Department of Physiotherapy, School of Medicine, College of Medicine and Health Science, University of Gondar, Gondar, Ethiopia; ^5^Department of Environmental and Occupational Health and Safety, College of Medicine and Health Sciences, Institute of Public Health, University of Gondar, Gondar, Ethiopia

**Keywords:** spatial variation, multilevel analysis, prenatal, HIV test uptake, Ethiopia

## Abstract

**Introduction:**

Human immunodeficiency virus (HIV) testing and counseling services are routine prenatal care services for the prevention of mother-to-child transmission of HIV. Although the prevalence of HIV infection is high among women, evidence suggests that the uptake of HIV testing during prenatal services in Ethiopia is scarce. Therefore, the aim of this study was to investigate individual- and community-level determinants and the spatial distribution of prenatal HIV test uptake in Ethiopia based on the 2016 Ethiopian Demographic and Health Survey.

**Methods:**

Data were accessed from the 2016 Ethiopian Demographic and Health Survey. A total weighted sample of 4,152 women aged 15–49 years who gave birth in the 2 years preceding the survey were included in the analysis. The Bernoulli model was fitted using SaTScan V.9.6 to identify cold-spot areas and ArcGIS V.10.7 to explore the spatial distribution of prenatal HIV test uptake. Stata version 14 software was used to extract, clean, and analyze the data. A multilevel logistic regression model was used to identify the individual- and community-level determinants of prenatal HIV test uptake. An adjusted odds ratio (AOR) with a corresponding 95% confidence interval (CI) was used to declare significant determinants of prenatal HIV test uptake.

**Results:**

The prevalence of HIV test uptake was 34.66% (95% CI: 33.23, 36.13%). The spatial analysis revealed that the distribution of prenatal HIV test uptake was significantly varied across the country. In the multilevel analysis, the following individual and community-level determinants were significantly associated with prenatal HIV test uptake: women who attained primary education (AOR = 1.47, 95% CI: 1.15, 1.87) and secondary and higher education (AOR = 2.03, 95% CI: 1.32, 3.11); women from middle (AOR = 1.46; 95% CI: 1.11, 1.91) and rich household wealth status (AOR = 1.81; 95% CI: 1.36, 2.41); those who had health facility visits in the last 12 months (AOR = 2.17; 95% CI: 1.77, 2.66); women who had higher (AOR = 2,07; 95% CI: 1.66, 2.59) and comprehensive HIV-related knowledge (AOR = 2.90; 95% CI: 2.09, 4.04); women who had moderate (AOR = 1.61; 95% CI: 1.27, 2.04), lower (AOR = 1.52; 95% CI: 1.15, 1.99), and no stigma attitudes (AOR = 2.67; 95% CI: 1.43, 4.99); those who had awareness of MTCT (AOR = 1.83; 95% CI: 1.50, 2.24); those from rural areas (AOR = 0.31; 95% CI: 0.16, 0.61); high community level of education for women (AOR =1.61; 95% CI: 1.04, 2.52); and those living in large central (AOR = 0.37; 95% CI: 0.15, 0.91) and small peripheral areas (AOR = 0.22; 95% CI: 0.08, 0.60).

**Conclusion:**

In Ethiopia, prenatal HIV test uptake had significant spatial variations across the country. Both individual- and community-level determinants were found to be associated with prenatal HIV test uptake in Ethiopia. Hence, the impact of these determinants should be recognized while developing strategies in “cold spot” areas of prenatal HIV test uptake to enhance prenatal HIV test uptake in Ethiopia.

## Introduction

Human immunodeficiency virus (HIV) remains a major global public health problem. Globally, an estimated 150,000 children were newly infected with HIV and 1.7 million children were living with HIV infection in 2020 (1). The vast majority of infections in children (0–14 years) were transmitted through mother-to-child transmission (MTCT) or vertical transmission during pregnancy, labor, or delivery or after delivery through breastfeeding from HIV-infected women to their babies (2). Without antiviral treatment, the risk of HIV transmission from infected mothers to their children is ~15–30% during pregnancy and labor, with an additional 5–20% becoming infected through prolonged breastfeeding (3). The intervention that aims to prevent MTCT of HIV is known as the prevention of mother-to-child transmission of HIV (PMTCT) (4).

Late maternal diagnosis of HIV and lack of antiretroviral treatment and prophylaxis are the major causes of MTCT for HIV (5). The World Health Organization recommended a prenatal HIV test for all pregnant women to prevent child infections *via* MTCT (6). In sub-Saharan Africa, the prenatal HIV test uptake is 60.7% (7), and in Ethiopia, only 34% of women take prenatal HIV tests at a national level (8).

Findings from previous literature revealed different factors associated with prenatal HIV test uptakes, such as maternal age (9), residence (7, 10), marital status (9), maternal education (11), maternal occupation (10), household wealth status (12, 13), media exposure (13, 14), distance to the health facility (11, 15), stigma (13), and limited knowledge of HIV (7).

Prenatal HIV testing and counseling is one of the programs for the PMTCT of HIV. Reducing new HIV infections among newborns has a huge benefit in terms of saving individual and societal costs as well as reducing the morbidity and mortality of children (16). However, the uptake of prenatal HIV testing in Ethiopia is low (8). In addition, the prevalence of prenatal HIV testing has significantly varied across the country (17, 18). Thus, the identification of spatial variation in prenatal HIV testing using geographical information systems (GISs) and spatial scan statistical analysis (SaTScan) is crucial for evidence-based public health interventions. Understanding the spatial variation of prenatal HIV test uptake will help to design more specific programs to promote access and use of HIV test services by pregnant women in “cold spot” areas. However, a single study was conducted on prenatal HIV test uptake in Ethiopia based on the nationally representative Ethiopian Demographic and Health Survey (EDHS) data (19), but this study failed to explore the spatial distribution of prenatal HIV test uptake in Ethiopia. Even though prenatal HIV test uptake depends on individual-level determinants as well as community-level determinants, a previous study has highlighted a range of individual-level factors associated with prenatal HIV test uptake, and there is a paucity of information on community-level determinants that affect prenatal HIV test uptake at the national level. Identifying the spatial variation and individual- and community-level determinants that act as enablers and/or barriers to prenatal HIV testing services is critical in combating the MTCT of HIV in Ethiopia. Therefore, this study aimed to assess the individual- and community-level determinants and spatial variation of prenatal HIV test uptake in Ethiopia using spatial and multilevel analysis.

## Methods

### Study design, period, and setting

Secondary data analysis was conducted based on the 2016 EDHS, which is a nationally representative survey conducted from 18 January 2016 to 27 June 2016. Ethiopia is found in the Horn of Africa with 1.1 million square kilometers of area and is the second most populous country in Africa with an estimated population of 100,613,986 people. Ethiopia's administrative structure is federally decentralized, with nine regional states (Afar; Amhara; Benishangul-Gumuz; Gambela; Harari; Oromia; Somali; Southern Nations, Nationalities and People's Region (SNNP); and Tigray) and two city administrations (Addis Ababa and Dire Dawa) (20).

### Data source, study population, and sampling procedure

The source of the data for this study was the 2016 EDHS. It is the fourth national-level survey implemented by the Central Statistical Agency (CSA) in the country. All reproductive-aged women who gave birth in the 2 years preceding the survey were included in the study. We used the women's dataset, which is an individual record file (IR file) for analysis. The data were weighted using an individual sampling weight for women (v005) divided by 1,000,000 before any statistical analysis to restore the survey's representativeness was conducted. Finally, a total weighted sample of 4,152 women was included in our study. A two-stage stratified cluster sampling technique was employed using the 2007 Population and Housing Census as a sampling frame in the 2016 EDHS. Stratification was employed by dividing each region into urban and rural areas, which yielded a total of 21 sampling strata. Samples of enumeration areas (EAs) were selected independently in each stratum in two stages. In the first stage of selection, 645 EAs (443 in rural areas and 202 in urban areas) were chosen with probability sampling proportional to the size of the EAs, with independent selection in each sampling stratum. A fixed number of 28 households per cluster were selected with an equal probability of systematic selection in the second stage. The detailed sampling procedure information has been included in the full EDHS 2016 report (8).

### Study variables

#### Outcome variable

The dependent variable of this study was prenatal HIV test uptake, which was a binary outcome variable coded as “0” if a woman did not take a prenatal HIV test or tested but had not received a test result, and “1” if a woman took the prenatal HIV test and received a test result.

#### Independent variables

The independent variables of this study were further classified into individual- and community-level variables. The individual-level variables used in the current study were maternal age, maternal education, maternal occupation, marital status, husband/partner education, husband/partner occupation, household wealth status, media exposure, HIV knowledge, awareness of MTCT, stigma toward people living with HIV, and visiting health facilities in the past 12 months. Six community-level variables, such as residence, region, distance to the health facility, community-level women's education, community-level poverty, and community-level media exposure, were also used as independent variables in our study. The three community-level variables, such as community-level women's education, community-level poverty, and community-level media exposure were created by aggregating individual-level variables since these variables are not directly found in the EDHS data and are categorized as low and high based on the median value.

### Operational definition

**HIV/AIDS-related knowledge:** This was measured by creating an index of correct responses to six questions included in the EDHS questionnaires, which are related to HIV prevention and misconceptions. The score was obtained by giving 0 points to those who answered the incorrect response and 1 point to respondents who knew the correct response. Participants were classified as having low HIV-related knowledge if they correctly answered ≤ 3 questions, high knowledge if they correctly answered 4–5 questions, and comprehensive knowledge if they correctly answered six questions (21).

**Awareness of MTCT:** This was built from the answers to three questions (MTCT during pregnancy, during delivery, and through breastfeeding); then the study participants were categorized as having an awareness if they answered all these three questions, and otherwise they do not have an awareness (22, 23).

**Risky sexual behavior** was assessed using a set of five questions and categorized as “no risk” (score 0), “some risk” (score 1), and “high risk” (score ≥ 2) (21).

**Stigma attitude toward people living with HIV:** This was generated using a set of six questions. For each item, the response was given a score of 0–1. The total score was 6, and it was categorized as “no stigma” (score 6), “low stigma” (score 4–5), “moderate stigma” (score 2–3), and “high stigma” (score ≤ 1) (21).

### Data management and statically analysis

Data extraction, cleaning, coding, and further analysis were done using Stata 14. Before conducting any statistical analysis, the data were weighted using sampling weight to restore the representativeness of the data sample as well as to obtain reliable statistical estimates.

In the DHS data, there is a hierarchical nature. Therefore, to account for cluster variability, a multilevel logistic regression model was fitted to estimate the association between the individual as well as community-level determinants and prenatal HIV test uptake. In this study, four models were fitted. These were as follows: Model I (a null model), which was fitted without independent variables, Model II (which examined only the effects of individual-level variables), Model III (considering only community-level variables), and Model IV (examined both individual- and community-level variables simultaneously). Random effect analysis (to measure variation between clusters) was computed using the intra-cluster correlation coefficient (ICC), proportional change in variance (PCV), and median odds ratio (MOR). The calculation for MOR and PCV is as follows;


(1) $$MOR=e^{0.95\sqrt{VA}}$$

(24)

where VA is the area-level variance


(2) $$PCV=\frac{Vnull-VA}{V\;null}*100\%$$

(24)

where Vnull = variance of the initial model and VA = variance of the final model.

Model comparison/fitness was assessed using deviance, and the model with the lowest deviance was used as the best-fitted model. Both bivariable and multivariable multilevel logistic regression analyses were done, and the crude odds ratios at 95% CI and those variables with a *p* ≤ 0.2 were considered for multivariable multilevel analysis. In the multivariable multilevel logistic regression analysis, those variables with *p* < 0.05 in the best-fitted model were declared significantly associated with prenatal HIV test uptake. Multi-collinearity was also checked using the variance inflation factor (VIF), which indicates that there was no multi-collinearity since all variables have a VIF < 5.

### Spatial analysis

A spatial analysis was applied to assess geographic variations in prenatal HIV test uptake cases among EDHS clusters. We computed the proportions of prenatal HIV test uptake cases in the survey for each cluster and then appended the latitude and longitude coordinates of the selected EAS in the 2016 EDHS survey. Both ArcGIS version 10.7 and SaTScan version 9.6 software were used for spatial analysis. Global spatial autocorrelation (Moran's I) was done to evaluate whether the pattern expressed is clustered, dispersed, or random across the study areas. It is a single output value in spatial statistics, which ranges from −1 to +1. The Moran's I index values close to −1 indicate that the spatial distribution of prenatal HIV test uptake is dispersed, whereas the Moran's I index values close to +1 indicate that the spatial distribution of prenatal HIV test uptake is clustered, and Moran's I value of 0 indicates that the spatial distribution of prenatal HIV test uptake is random. In addition, the statistically significant Moran's I (*p* < 0.05) indicates the spatial clustering of prenatal HIV test uptake.

Getis-Ord Gi^*^ statistics were calculated to determine how spatial autocorrelation differs across study locations. Statistical output with a high GI^*^ denotes a “hot spot,” whereas a low GI^*^ suggests a “cold spot” (25). The spatial interpolation was done to predict prenatal HIV test uptake on the un-sampled EAs in the country based on sampled measurements. The ordinary Kriging spatial interpolation was selected for this study to predict the prevalence of prenatal HIV test uptake on the unobserved EAs based on the observed measurement because it incorporates spatial autocorrelation and statistically optimizes the weight (26). The spatial interpolation was performed under the assumption that spatially distributed objects are spatially correlated, and that objects that are close together are more likely to have similar properties (27, 28).

In the spatial scan statistical analysis, the Bernoulli-based model was employed to detect statistically significant spatial clusters of prenatal HIV test uptake using Kuldorff's SaTScan version 9.6 statistical software. The Bernoulli model required information about the location of a set of cases and controls using cases, controls, and geographic coordinates data. Pregnant women who are tested for HIV during the prenatal period were taken as cases, whereas those who are not tested for HIV were considered as controls to fit the Bernoulli model. The default maximum spatial cluster size of < 50% of the population was used as an upper limit, allowing for the detection of both small and large clusters while ignoring clusters that contained more than the maximum limit. To determine the statistical significance of clustering, the Z-score was computed, and the *p*-value was used to determine if the number of observed cases within the potential cluster was significant or not (29). Moreover, this spatial analysis method has been used in various studies (30–32).

### Ethical consideration

Since the study was a secondary data analysis of publicly available survey data from the MEASURE DHS program and geographical coordinate data, ethical approval and participant consent were not necessary for this particular study. We obtained permission to download and use the data from DHS Program at http://www.measuredhsprogram.com. There were no names of individuals or household addresses in the data file.

## Results

### Sociodemographic characteristics of study participants

A total of 4,152 women aged 15–49 years who gave birth in the 2 years preceding the survey were included in the analysis. Half (50.7%) of the respondents were aged 25–34 years, and the majority (94.11%) of the respondents were married. The majority of the participants, 60.2% and 58.7% had no formal education and employment, respectively. Regarding media exposure, nearly two-thirds (65.8%) of the respondents were unexposed. Approximately 53% of the respondents had facility visits in the last 12 months. Out of the total participants, 2,228 (53.7%) had awareness of MTCT and 3,354 (80.8%) reported no risky sexual behavior. The majority (87.9%) of the respondents were rural residents, and a half (50.8%) of the respondents lived in areas with low community-level women's education (Table 1).

**Table 1:** Sociodemographic characteristics of study participants in a study of spatial variation and its determinants of prenatal HIV test uptake in Ethiopia based on the 2016 EDHS.

**Variables**	**Category**	**Weighted frequency (%)**
Age	15–24	1,222 (29.4)
	25–34	2,106 (50.7)
	35–49	824 (19.9)
Marital status	Never married	30 (0.7)
	Married	3,907 (94.1)
	Divorced/widowed/separated	214 (5.2)
Religion	Orthodox Christian	1,414 (34.1)
	Muslim	1,724 (41.5)
	Protestant	861 (20.8)
	Others	153 (3.7)
Maternal education status	No formal education	2,498 (60.2)
	Primary education	1,288 (31.0)
	Secondary and above education	366 (8.8)
Husband education	No formal education	1,791 (45.3)
	Primary education	1,588 (40.2)
	Secondary and above education	575 (14.5)
Women's occupation	Unemployed	2,436 (58.7)
	Employee	1,715 (41.3)
Husband occupation	Unemployed	314 (7.9)
	Employee	3,640 (92.1)
Wealth index	Poor	1,876 (45.2)
	Middle	873 (21.0)
	Rich	1,403 (33.8)
Media exposure	Unexposed	2,731 (65.8)
	Exposed	1,421 (34.2)
Visiting health facility	No	1,952 (47.0)
	Yes	2,200 (53.0)
HIV related knowledge	Low	1,786 (43.0)
	High	1,814 (43.7)
	Comprehensive	551 (13.3)
Awareness of MTCT	No	1,923 (46.3)
	Yes	2,229 (53.7)
Stigma toward people living with HIV/AIDS	No stigma	120 (2.9)
	Low	957 (23.1)
	Moderate	1,525 (36.7)
	High	1,549 (37.3)
Risky sexual behavior	No risk	3,354 (80.8)
	Low risk	731 (17.6)
	High risk	67 (1.6)
Desire of children	Unwanted	1,374 (33.1)
	Wanted	2,778 (66.9)
Residence	Urban	503 (12.1)
	Rural	3,649 (87.9)
Region	Metropolis	134 (3.2)
	Large centrals	3,752 (90.4)
	Small peripherals	266 (6.4)
Distance to health facility	Not a big problem	1,642 (39.5)
	Big problem	2,510 (60.5)
Community level media exposure	Low	2,033 (49.0)
	High	2,119 (51.0)
Community level poverty	Low	2,503 (60.3)
	High	1,649 (39.7)
Community level women's education	Low	2,110 (50.8)
	High	2,042 (49.2)

### Spatial analysis results

#### Spatial distribution of prenatal HIV test uptake in Ethiopia

**Figure 1:**
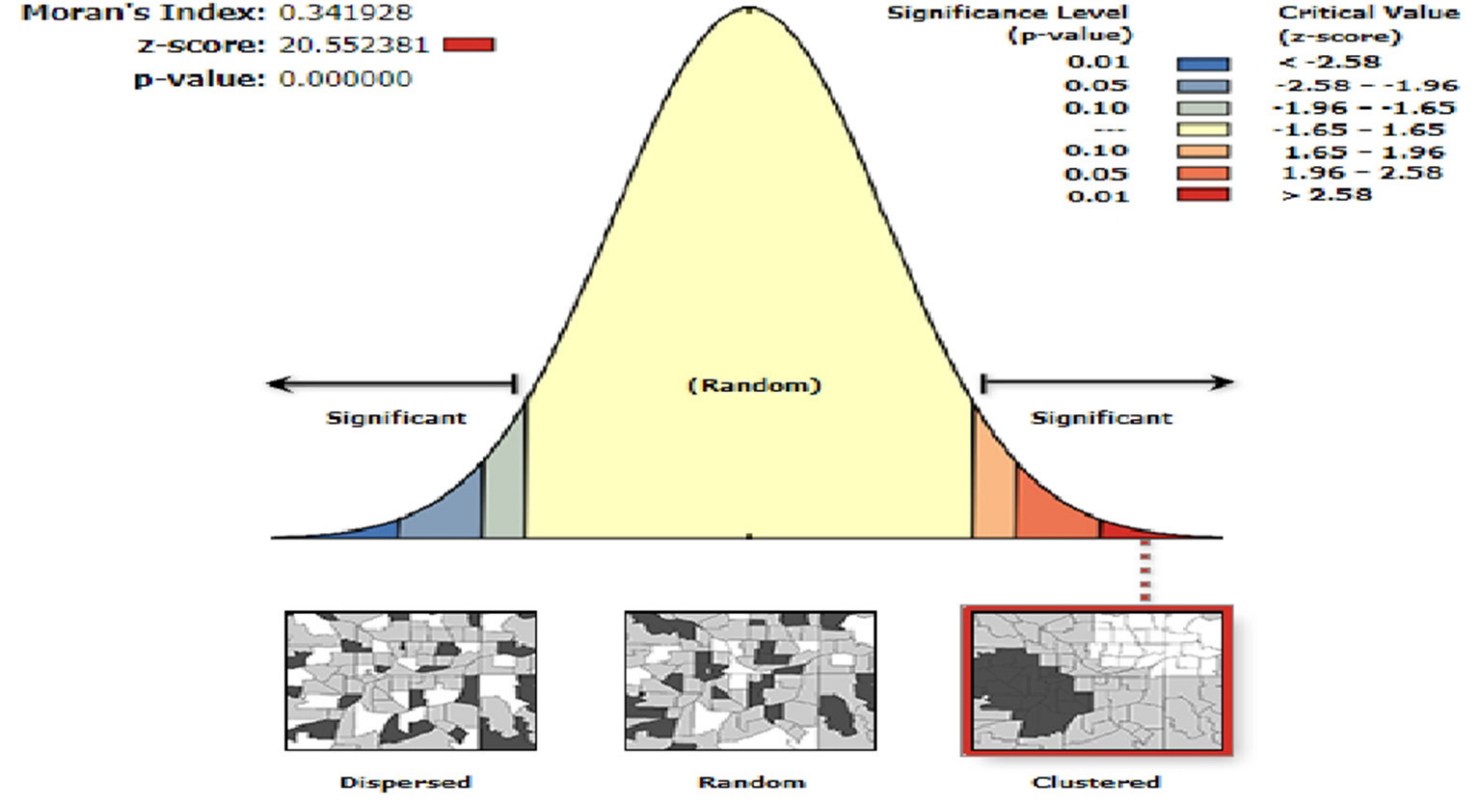
Global autocorrelation of prenatal HIV test uptake in Ethiopia, 2016.

**Figure 2:**
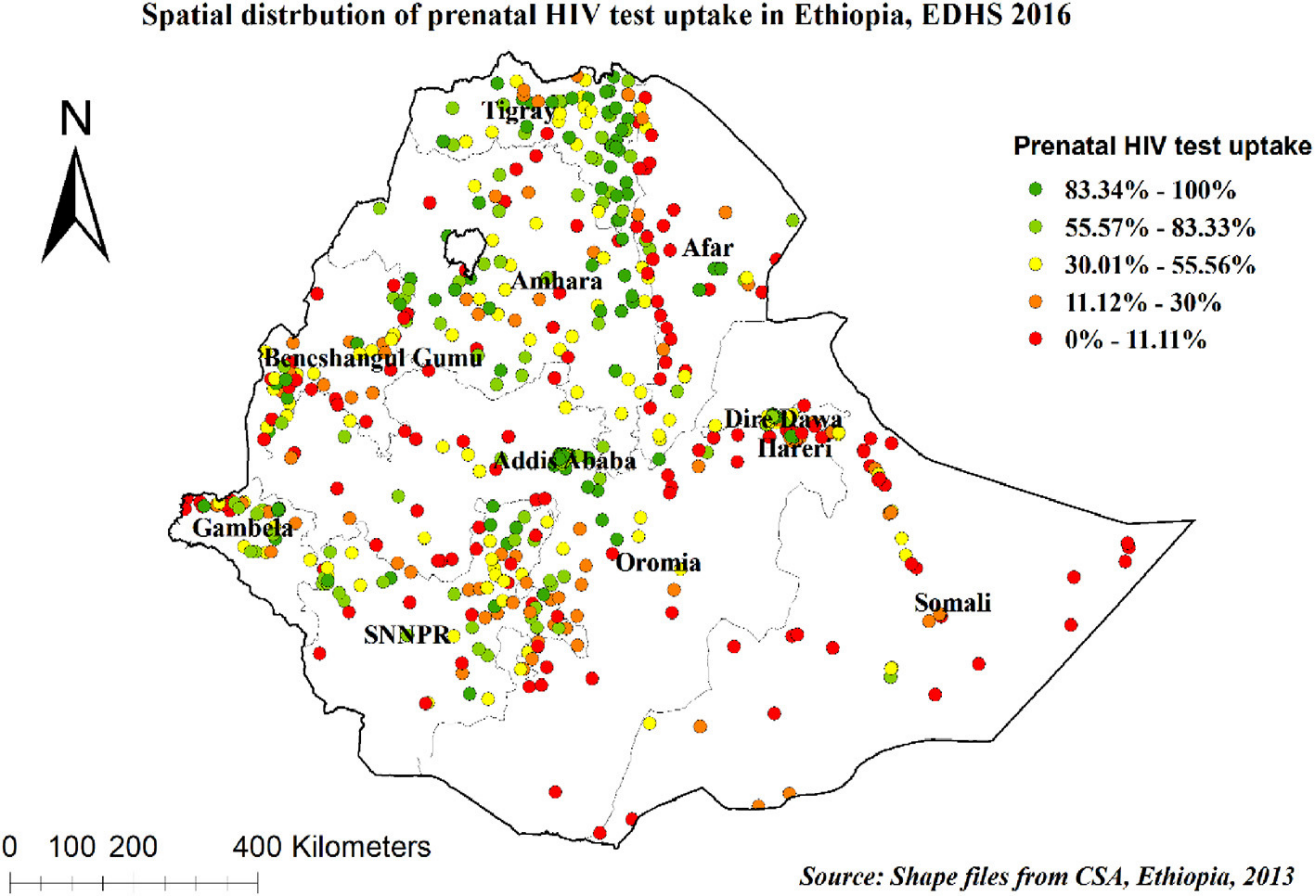
Spatial distribution of prenatal HIV test uptake in Ethiopia, 2016.

The spatial autocorrelation analysis revealed that the spatial distribution of prenatal HIV test uptake significantly varied across Ethiopia (Global Moran's I value of 0.34 (*p* < 0.001)), which means that there was a high prenatal HIV test uptake in some areas and there was a low prenatal HIV test uptake in some areas. A Z-score of 20.55 indicates that there is less than a 1% likelihood that this clustered pattern could be due to random chance (Figure 1). In our study, areas with a high prevalence of prenatal HIV test uptake were detected in Addis Ababa, Dire Dawa, and Tigray regions. In contrast, areas with a low prevalence of prenatal HIV test uptake were identified in Afar, Somali, Benishangul-Gumuz, and Gambella regions (Figure 2).

#### Cold spot analysis and spatial scan statistical analysis of prenatal HIV test uptake in Ethiopia

**Figure 3:**
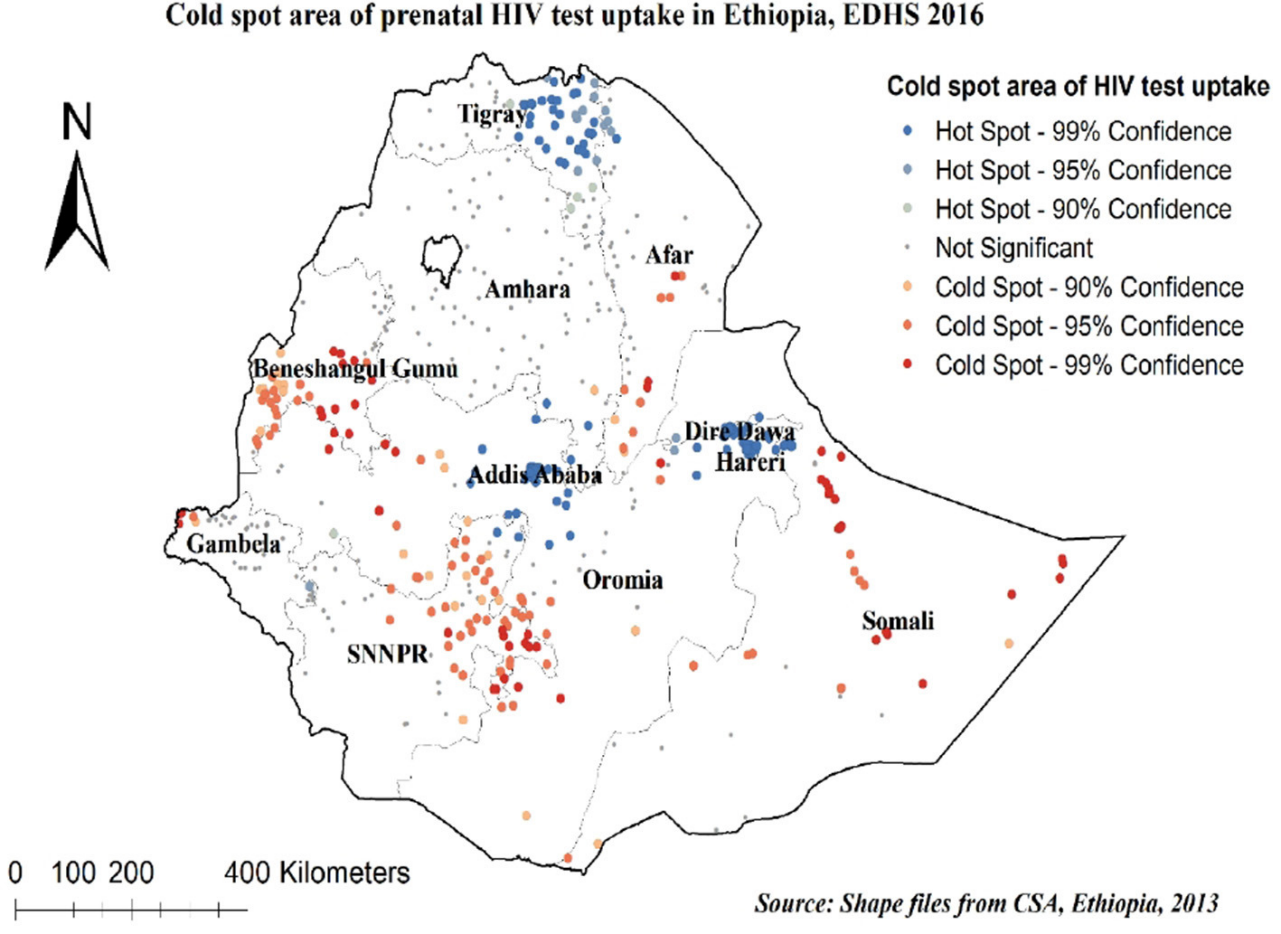
Hot spot and cold spot identifications of prenatal HIV test uptake in Ethiopia, 2016.

In cold spot analysis of prenatal HIV test uptake, a significant cluster was detected. Cold spot areas of prenatal HIV test uptake were found in Somali, Afar, Northeast SNNPR, Northwest Gambela, West Oromia, and most parts of the Benishangul-Gumuz regions, while hot spot areas of prenatal HIV test uptake were found in Addis Ababa, Dire Dawa, Harari, and East Tigray regions (Figure 3).

**Figure 4:**
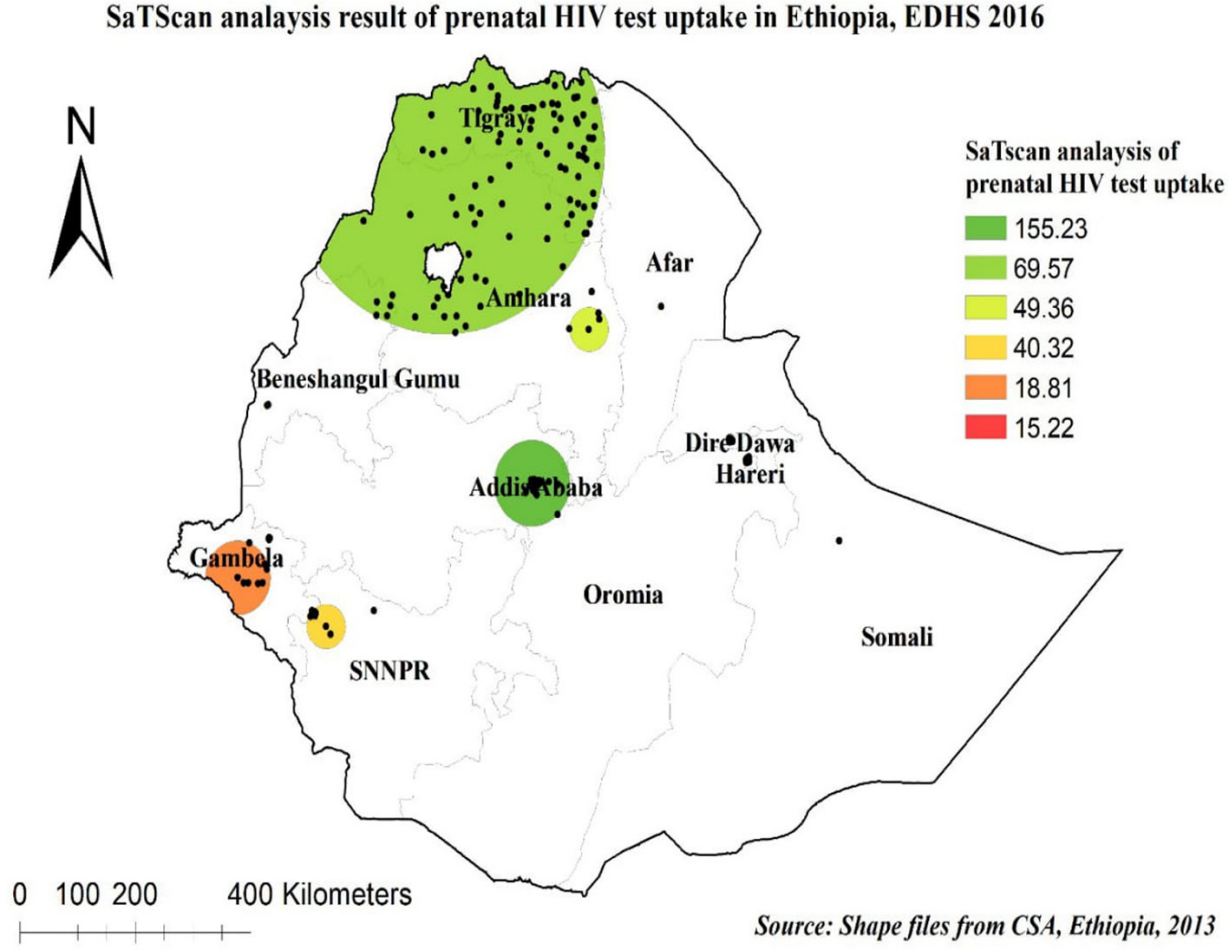
SaTScan analysis of prenatal HIV test uptake in Ethiopia, 2016.

In the spatial scan statistical analysis, a total of 231 significant clusters of prenatal HIV test uptake were identified. Of these, 58 were most likely (primary) clusters and 173 were secondary clusters. The primary clusters were located in the entire Addis Ababa city at 9.025638 N, 38.717479 E geographical location with a 65.36 km radius, a relative risk (RR) of 2.51, and log-likelihood ratio (LLR) of 155.23, at *p* < 0.0001 (Figure 4, Table 2). It showed that women inside the spatial window had a 2.51 times higher likelihood of being tested for prenatal HIV than women outside the spatial window.

**Table 2:** SaTScan analysis result of prenatal HIV test uptake in Ethiopia based on the 2016 EDHS.

**Cluster**	**Enumeration area (cluster) identified**	**Coordinate/radius**	**Population**	**Case**	**RR**	**LLR**	***P*-value**
1 (58)	100, 31, 107, 339, 626, 108, 11, 195, 314, 635, 369, 59, 305, 487, 532, 645, 414, 159, 463, 274, 582, 608, 170, 144, 15, 110, 153, 112, 293, 145, 225, 639, 247, 19, 464, 264, 61, 451, 539, 155, 428, 509, 330, 261, 475, 560, 211, 287, 252, 402, 90, 147, 236, 83, 353, 303, 40, 290	(9.025638 N, 38.717479 E)/65.36 km	222	210	2.51	155.23	< 0.0001
2(101)	612, 296, 504, 258, 253, 638, 322, 583, 80, 188, 312, 640, 340, 268, 98, 279, 152, 425, 255, 181, 327, 528, 78, 628, 551, 584, 597, 156, 292, 400, 636, 163, 590, 579, 81, 199, 52, 575, 84, 355, 158, 481, 542, 512, 45, 538, 169, 479, 604, 461, 132, 424, 89, 73, 430, 431, 66, 392, 237, 226, 94, 550, 129, 160, 259, 516, 598, 300, 136, 605, 220, 456, 404, 143, 341, 384, 382, 167, 623, 602, 627, 415, 361, 99, 449, 298, 413, 196, 421, 79, 403, 541, 401, 117, 192, 128, 97, 386, 442, 429, 351	(13.545336 N, 37.308758 E)/276.96 km	660	407	1.68	69.57	< 0.0001
3 (31)	101, 140, 190, 546, 363, 467, 644, 43, 606, 224, 390, 385, 111, 282, 27, 185, 493, 444, 514, 5, 151, 631, 471, 273, 535, 519, 74, 380, 613, 352, 202	(9.620244 N, 41.840029 E) /7.20 km	103	90	2.20	49.36	< 0.0001
4 (31)	396, 387, 238, 157, 60, 257, 397, 534, 56, 44, 228, 393, 28, 329, 321, 383, 418, 194, 614, 240, 587, 443, 29, 500, 179, 495, 173, 483, 133, 381, 115	(9.329465 N, 42.127591 E) /5.22 km	116	94	2.04	40.32	< 0.0001
5 (6)	221, 291, 549, 469, 47, 231	(8.243521 N, 34.585152 E) /1.66 km	21	21	2.46	18.81	< 0.0001
6 (4)	18, 345, 611, 616	(11.120147 N, 39.621725 E) /33.47 km	17	17	2.46	15.21	< 0.0001

#### Spatial interpolation of prenatal HIV test uptake

**Figure 5:**
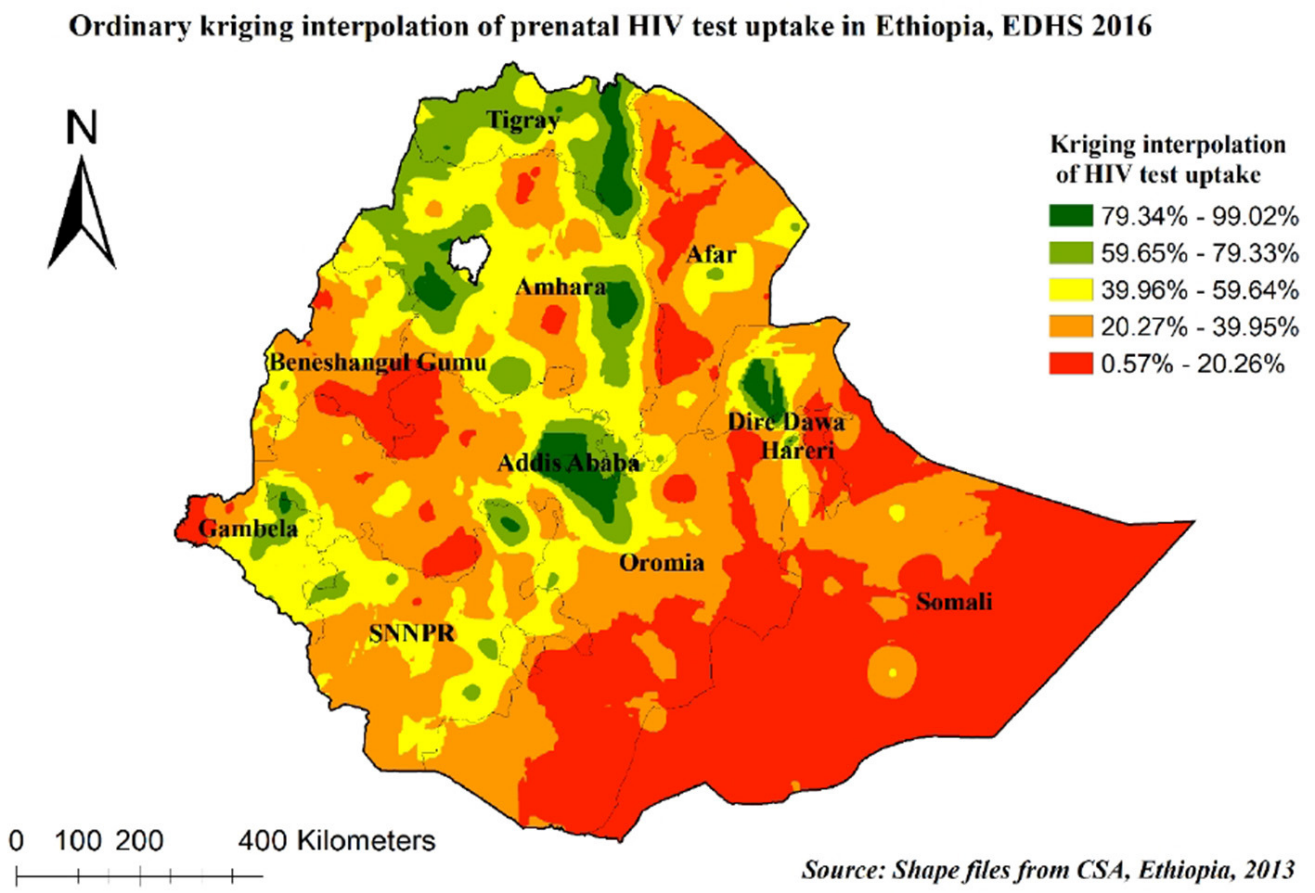
Kriging interpolations of prenatal HIV test uptake in Ethiopia, 2016.

The spatial interpolated analysis results predicted the unobserved areas in Ethiopia based on the observed measurements. The green color indicates predicted high prenatal HIV test uptake and the red color indicates the predicted low prenatal HIV test uptake areas. In this study, the Kriging interpolation map predicted the highest prevalence of prenatal HIV test uptake to be detected in Addis Ababa, Dire Dawa, Harari, Amhara, and Tigray regions. The predicted low prevalence of prenatal HIV test uptake was identified in the Southeast Oromia, Afar, Somali, Benishangul-Gumuz, and West Gambela regions (Figure 5).

### Individual- and community-level determinants of prenatal HIV test uptake

#### Measures of variation (random effect) and model fitness

As shown in Table 3, the null model (Model I) revealed a statistically significant variation in prenatal HIV test uptake between clusters. The value of ICC in the null model was 0.553, in which 55.3% of the variations of prenatal HIV test uptake were attributed to differences between clusters, but the rest, 44.7%, were due to individual differences. In this study, the highest MOR (6.80) was revealed in the null model, which means that there was a variation in the uptake of prenatal HIV testing between clusters. If we randomly select pregnant women from different clusters, those from the cluster with higher rates of prenatal HIV test uptake had 6.80 times higher odds of taking a prenatal HIV test as compared with those pregnant women from a cluster with lower rates of prenatal HIV test uptake. Moreover, the PCV value in the final model (Model IV) was 43%, which indicates that the variation in the uptake of prenatal HIV tests was explained by the final model (both the individual- and community-level determinants). A deviance test was done to check model comparison/fitness, and the final model had the lowest deviance (3,486) and was taken as the best-fitted model (Table 3).

**Table 3:** Multivariable multilevel logistic regression analysis of individual- and community-level determinants of prenatal HIV test uptake in Ethiopia based on the 2016 EDHS.

**Variables**	**Model 1**	**Model 2**	**Model 3**	**Model 4**
		**AOR (95%CI)**	**AOR (95%CI)**	**AOR (95%CI)**
**Age**				
15–24	-	1	-	1
25–34	-	0.99 (0.78, 1.27)	-	0.93 (0.73, 1.19)
≥35	-	0.88 (0.63, 1.21)	-	0.85 (0.61, 1.17)
**Women's educational status**				
No formal education	-	1	-	1
Primary education	-	1.64 (1.29, 2.08)	-	1.47 (1.15, 1.87)^**^
Secondary and above education	-	2.84 (1.88, 4.29)	-	2.03 (1.32, 3.11)^**^
**Women's occupation**				
Unemployed	-	1	-	1
Employee	-	1.14 (0.93, 1.41)	-	1.13 (0.92, 1.40)
**Wealth index**				
Poor	-	1	-	1
Middle	-	1.64 (1.26, 2.15)	-	1.46 (1.11, 1.91)^**^
Rich	-	2.55 (1.95, 3.34)	-	1.81 (1.36, 2.41)^***^
**Media exposure**				
Unexposed	-	1	-	1
Exposed	-	1.28 (1.02, 1.62)	-	1.10 (0.87, 1.39)
**Visiting health facility**				
No	-	1	-	1
Yes	-	2.23 (1.82, 2.73)	-	2.17 (1.77, 2.66)^***^
**HIV related knowledge**				
Low	-	1	-	1
High	-	2.19 (1.75, 2.74)	-	2.07 (1.66, 2.59)^***^
Comprehensive	-	3.13 (2.26, 4.35)	-	2.90 (2.09, 4.04)^***^
**Stigma toward PLWH**				
No	-	2.85 (1.55, 5.25)	-	2.67 (1.43, 4.99)^**^
Low	-	1.68 (1.28, 2.20)	-	1.52 (1.15, 1.99)^**^
Moderate	-	1.70 (1.34, 2.16)	-	1.61 (1.27, 2.04)^***^
High	-	1	-	1
**Awareness of MTCT**				
No	-	1	-	1
Yes	-	1.83 (1.50, 2.23)	-	1.83 (1.50, 2.24)^***^
**Desire of children**				
Unwanted	-	1	-	1
Wanted	-	1.16 (0.93, 1.45)	-	1.18 (0.94, 1.47)
**Community level variables**				
**Residency**				
Urban	-	-	1	1
Rural	-	-	0.17 (0.09, 0.33)	0.31 (0.16, 0.61)^**^
**Region**				
Metropolis	-	-	1	1
Large centrals	-	-	0.34 (0.14, 0.80)	0.37 (0.15, 0.91)^*^
Small peripherals	-	-	0.13 (0.05, 0.34)	0.22 (0.08, 0.60)^**^
**Distance to health facility**				
Not a big problem	-	-	1	1
Big problem	-	-	0.72 (0.58, 0.89)	0.80 (0.64, 1.01)
**Community level poverty**				
Low	-	-	1	1
High	-	-	0.45 (0.28, 0.70)	0.71 (0.44, 1.14)
**Community level media exposure**				
Low	-	-	1	1
High	-	-	1.71 (1.10, 2.66)	1.42 (0.89, 2.24)
**Community level women's education**				
Low	-	-	1	1
High	-	-	2.41 (1.57, 3.71)	1.61 (1.04, 2.52)^*^
**Random effect**				
VA	4.07	2.43	2.40	2.31
ICC	0.553	0.424	0.422	0.413
MOR	6.80	4.40	4.36	3.93
PCV	Reff	0.40	0.41	0.43
**Model comparison**				
Deviance	4,122	3,564	3,848	3,486
Mean VIF	-	1.33	1.89	1.62

^*^P < 0.05, ^**^P < 0.01, ^***^P < 0.001.

ICC, Inter cluster correlation coefficient; MOR, median odds ratio; PCV, proportional change in variance; AOR, adjusted odds ratio; CI, confidence interval; VIF, variance inflation factor.

#### Measure of association (fixed-effect) analysis

The overall prevalence of HIV test uptake was 34.66% (95% CI: 33.23, 36.13%). In the multilevel logistic regression analysis, both individual- and community-level determinants were significantly associated with prenatal HIV test uptake. Individual-level determinants such as women's education, household wealth status, visiting health facilities in the last 12 months, HIV-related knowledge, stigma toward people living with HIV, and awareness of MTCT were found to be associated with prenatal HIV test uptake. Community-level determinants, such as residence, region, and community-level women's education, were significantly associated with the outcome variable.

Pregnant women who attained primary education and secondary and above education had 1.47 (AOR = 1.47, 95% CI: 1.15, 1.87) and 2.03 (AOR = 2.03, 95% CI: 1.32, 3.11) times higher odds of prenatal HIV test uptake than those who did not attain formal education, respectively. Women from middle household wealth status and rich household wealth status had 1.46 (AOR = 1.46; 95% CI: 1.11, 1.91) and 1.81 (AOR = 1.81; 95% CI: 1.36, 2.41) times higher chances of being tested for HIV as compared with their counterparts, respectively. The odds of prenatal HIV test uptake were 2.17 times (AOR = 2.17; 95% CI: 1.77, 2.66) higher among pregnant women who had visited health facilities in the previous 12 months as compared to their counterparts. The odds of prenatal HIV test uptake among pregnant women with higher and comprehensive HIV related knowledge were 2.07 times (AOR = 2.07; 95%CI: 1.66, 2.59) and 2.90 times (AOR = 2.90; 95%CI: 2.09, 4.04) higher than among pregnant women with low HIV related knowledge, respectively.

Having a stigmatizing attitude toward people living with HIV/AIDS was significantly associated with prenatal HIV test uptake. Compared to pregnant women who had higher stigma attitudes, those who had moderate, lower, and no stigma attitudes were 1.61 (AOR = 1.61; 95% CI: 1.27, 2.04), 1.52 (AOR = 1.52; 95% CI: 1.15, 1.99), and 2.67 times (AOR = 2.67; 95% CI: 1.43, 4.99) more likely to be tested for prenatal HIV test, respectively. Pregnant women who were aware of MTCT had 1.83 times (AOR = 1.83; 95% CI: 1.50, 2.24) higher odds of HIV testing than their counterparts.

Regarding community-level determinants, the odds of prenatal HIV test uptake among pregnant women residing in rural areas were 69% (AOR = 0.31; 95% CI: 0.16, 0.61) less likely than pregnant women residing in urban areas. Pregnant women living in large central and small peripheral areas had 63% (AOR = 0.37; 95% CI: 0.15, 0.91) and 78% (AOR = 0.22; 95% CI: 0.08, 0.60) lower odds of prenatal HIV test uptake than those who are living in metropolitan areas respectively. Moreover, pregnant women residing in communities with a high proportion of educated women had 1.6 times (AOR =1.61; 95% CI: 1.04, 2.52) higher prenatal HIV test uptake as compared with pregnant women residing in communities with a low proportion of educated pregnant women (Table 3).

## Discussion

This study assessed the spatial distribution and individual- and community-level predictors associated with prenatal HIV test uptake in Ethiopia based on the nationally representative EDHS data. The prevalence of prenatal HIV test uptake in Ethiopia was found to be 34.66% (95% CI: 33.23, 36.13%), with marked spatial heterogeneity. The spatial distribution of prenatal HIV test uptake varied significantly across the country. In Somali, Afar, Northeast SNNPR, Northwest Gambela, West Oromia, and most parts of the Benishangul-Gumuz regions, significant cold spot areas with a low prevalence of prenatal HIV test uptake were identified. The possible explanation might be due to the spatial clustering of HIV infection among reproductive-age women in the country (33). This could also be attributed to the disparity in the availability and inaccessibility of maternal health services in the border areas of regions (34). Furthermore, HIV-related knowledge is suboptimal in the Somali, Afar, Benishangul-Gumuz, and Gambela regions, resulting in low prenatal HIV test uptake (35). This finding suggests that policymakers and public health planners should design effective public health interventions to enhance prenatal HIV test uptake in these significant cold spot areas where prenatal HIV test uptake is low.

In the multilevel analysis, different individual and community determinants were significantly associated with prenatal HIV test uptake. Among the individual-level determinants, it was found that the odds of prenatal HIV test uptake among pregnant women who have attained primary, secondary and above education were higher than those pregnant women who had not attained formal education. This result is consistent with other studies conducted elsewhere (7, 11, 36, 37). The possible justification might be that women's educational status plays a key role in their empowerment, financial wellbeing, and capacity to access healthcare services (38). Women's education also promotes women's health-seeking behavior through its positive role in health awareness (39). In addition, educated women are more likely to be able to acquire information regarding the health risks associated with pregnancy and increase their health knowledge, which enables them to take preventive measures and reach out for professional help such as counseling and tests for HIV (40).

Based on our study, household wealth status is a very important determinant of whether a pregnant woman takes a prenatal HIV test or not. The findings revealed that pregnant women from middle and rich household wealth status had increased odds of prenatal HIV test uptake as compared with pregnant women with a poor wealth status. This result is supported by prior studies (11, 12). This might be due to better economic status, which may increase healthcare-seeking behavior and autonomy in healthcare decision-making. In addition, despite maternal services being free of charge in Ethiopia, distances to healthcare facilities and the capability of paying the required transportation costs to access health services could be challenges for poorer women (41, 42).

Consistent with previous studies (9, 43, 44), this study revealed that visiting a health facility was a significant predictor of prenatal HIV test uptake. The likelihood of testing for HIV during the prenatal period was higher among pregnant women who visited health facilities in the last 12 months than those who did not visit health facilities. The possible justification might be that women who visited health facilities had a chance to get information on HIV testing for the prevention of MTCT and obtain services because of the initiative of healthcare providers (45).

The odds of prenatal HIV testing among pregnant women who had higher and comprehensive HIV-related knowledge were higher than those who had low HIV-related knowledge. This finding is congruent with studies conducted elsewhere (46–48). This might be explained by the fact that pregnant women with higher and comprehensive HIV-related knowledge have a better understanding of both the transmission and prevention of HIV/AIDS from mother to child, which helps them seek and test for prenatal HIV to prevent mother-to-child transmission of HIV/AIDS (49, 50).

This study has identified that stigma toward people living with HIV/AIDS was significantly associated with HIV testing during the prenatal period. Women who had moderate to no stigma attitudes were more likely to be tested for HIV during the prenatal period as compared with those who had higher stigma attitudes. This result aligns with different studies conducted in Ethiopia (17, 51) and Kenya (52, 53). This could be because stigma might reduce the uptake of HIV testing. Reducing stigma and discrimination is a critical gateway to increasing the uptake of HIV testing and PMTCT services (54).

The other determinant that was significantly associated with prenatal HIV test uptake was awareness of MTCT. Women who had awareness about MTCT had higher odds of being tested for HIV during the prenatal period compared with those who had no awareness about MTCT. This result is consistent with studies conducted in Northwest Ethiopia (17), Tanzania (55), and Brazil (56). This could be because to women who were aware of MTCT had a higher perceived benefit of HIV counseling and testing to prevent MTCT of HIV (18).

Among the community-level determinants, women from high community-level education had higher odds of prenatal HIV testing. This finding is supported by a previous study (9). The possible reason is that education may enhance health-seeking behaviors and health service utilization through sharing experiences about where and when to get the available services as well as the advantages and risks of accessing services from the community (57–59). In addition, prenatal HIV test uptake increased among educated women due to improvements in women's decision-making power, knowledge, and utilization of health care services (60, 61).

In this study, place of residence was significantly associated with prenatal HIV test uptake. Consistent with other studies conducted in Mozambique (10), Zimbabwe (13), East Africa (11), and sub-Saharan Africa (7), in this study, women residing in rural areas had reduced odds of prenatal HIV testing compared with pregnant women residing in urban areas. The possible explanation might be that women residing in rural areas have less health awareness, financial capacity, and transportation facilities, and lack access to antenatal and HIV testing centers (62). Rural residents are underutilizing essential healthcare services when compared to their urban counterparts (63).

In multilevel analyses of this study, pregnant women living in large central and small peripheral areas had lower odds of prenatal HIV test uptake than pregnant women living in metropolitan areas. The possible reason might be the disparity in the accessibility of maternal health services in the country (34, 64). Furthermore, pregnant women who live in the metropolis area may be better educated than their counterparts, which allows them to use prenatal HIV tests because education plays a significant role in maternal health service utilization (64).

Finally, this study used a national-based dataset with spatial analysis and a multilevel logistic regression model to describe the spatial variation and identify the associated factors of prenatal HIV test uptake in Ethiopia. The use of a multilevel logistic regression model is paramount to getting a reliable standard error because the model accounts for the correlated nature of DHS data. Therefore, we hope this study will add something to the existing knowledge about this problem, especially the factors affecting prenatal HIV test uptake in Ethiopia that will enable appropriate and specific interventions by the concerned bodies to be taken to tackle the MTCT of HIV. However, our study was not without limitations. Since this survey relies on respondents' self-reporting, there may be the possibility of recall bias and social desirability bias. The GPS data (latitude and longitude) taken at EA were displaced by 10 km in rural areas and 5 km in urban areas for the privacy issue; this could bias our spatial result.

## Conclusion

In Ethiopia, prenatal HIV test uptake had spatial variations across the country. Statistically significant cold spot areas (low prevalence of prenatal HIV test uptake) of prenatal HIV test uptake were found in Somali, Afar, Northeast SNNPR, Northwest Gambela, West Oromia, and most parts of the Benshangul-Gumuz regions. Individual-level determinants, such as women's educational status, wealth index, visiting health facility, HIV-related knowledge, awareness of MTCT, and stigma attitude toward PLWH, and community-level determinants, such as residency, region, and community-level women's education, were significantly associated with prenatal HIV test uptake. For PMCT interventions to be successful, urgent attention should be focused on maternal education, increasing HIV-related knowledge, addressing stigma attitudes toward people living with HIV, and targeting the “cold spot” of prenatal HIV test uptake to enhance its utilization in the country.

## Data availability statement

All relevant data are within the article/supplementary material, further inquiries can be directed to the corresponding author.

## Ethics statement

Since the study was a secondary data analysis of publicly available survey data from the MEASURE DHS program and geographical coordinate data, ethical approval and participant consent were not necessary for this particular study. We obtained permission to download and use the data from DHS Program at http://www.measuredhsprogram.com. There were no names of individuals or household addresses in the data file.

## Author contributions

The conception of the study, design of the study, acquisition of data, analysis, and interpretation of data were done by NT, DB, and FA. Data curation, drafting the article, revising it critically for intellectual content, validation, and final approval of the version to be published were done by NT, DB, MA, MG, ME, and FA. All authors read and approved the final manuscript.
